# Sublingual misoprostol versus manual vacuum aspiration for treatment of incomplete abortion in Nigeria: a randomized control study

**DOI:** 10.11604/pamj.2022.41.90.29364

**Published:** 2022-02-01

**Authors:** Vincent Chinedu Ani, Joseph Tochukwu Enebe, Cyril Chukwudi Dim, Ngozi Regina Dim, Benjamin Chukwuma Ozumba

**Affiliations:** 1 Departments of Obstetrics and Gynaecology, College of Health Sciences Nnamdi Azikiwe University Nnewi Campus, Anambra State, Nigeria,; 2 Department of Obstetrics and Gynaecology, Enugu State University of Science and Technology, College of Medicine/ Teaching Hospital, Parklane, Enugu, Nigeria,; 3 Department of Obstetrics and Gynaecology, College of Medicine, University of Nigeria, Ituku-Ozalla, P.M.B. 01129, Enugu, Nigeria,; 4 Institute of Maternal and Child Health, College of Medicine, University of Nigeria, Ituku-Ozalla Campus, Enugu, Nigeria,; 5 Department of Radiation Medicine, College of Medicine, University of Nigeria Ituku-Ozalla Campus, Enugu, Nigeria

**Keywords:** Sublingual misoprostol, post-abortion care, complete abortion, manual vacuum aspiration

## Abstract

**Introduction:**

single-dose of sublingual misoprostol 400mcg with the participant followed-up at the gynecology clinic one week after with an ultrasound scan for the completeness of the uterine evacuation. Objective: to compare the effectiveness of single-dose sublingual misoprostol to manual vacuum aspiration in the treatment of incomplete spontaneous abortion in Enugu, Nigeria.

**Methods:**

the primary outcome measure was the incidence of complete uterine evacuation (complete abortion) after one week of treatment while the secondary outcome measures included incidence, types, and tolerability of treatment side effects as well as participants' satisfaction with the treatment received.

**Results:**

two hundred and three participants who met the study criteria and completed the study were randomised into the intervention group (n=102) received single-dose sublingual misoprostol 400mcg and the control group (n= 101) received manual vacuum aspiration. Incidence of complete abortion was 86.3% for the misoprostol group and 100.0% for the control group, RR = 0.86, (CI 95%: 0.80 - 0.93), p <0.001. The most common side effect was abdominal pain with an incidence of 27.5% versus 48.55 for the misoprostol and control groups respectively (p = 0.002). Most participants in each group (81.1% versus 77.6% for the misoprostol and control groups respectively) considered the side effects as tolerable. The mean visual analogue scale score for maternal satisfaction was higher in the misoprostol group (86.7 ± 14.11) than the control group (81.36 ± 11.10), p < 0.001.

**Conclusion:**

the treatment of incomplete spontaneous abortion with single-dose sublingual misoprostol 400mcg produced a high rate of complete abortion among women in Enugu, Nigeria. Despite having a lower complete abortion rate, maternal satisfaction was higher when compared with women that had manual vacuum aspiration of the uterus. Trial registration: trial registration number - PACTR202009857889210, date of registration - September 23^rd^, 2020. Retrospectively registered.

## Introduction

Abortion which can be spontaneous or induced contributes up to 13.2% of all maternal deaths globally [1]. It is referred to as incomplete when the products of conception are retained within the uterine cavity and may lead to complications such as hemorrhage, infection, renal failure, and death. These products of conception can be detected on ultrasound examination. It has been established that abortion-related death is especially prevalent in low resource settings, wherever abortion laws are restrictive or when access to safe abortion services is difficult [2]. For instance, in Nigeria, the Federal Ministry of Health (FMoH) attributes 11% of maternal deaths to complications of abortion [3]. Therefore, to address complications related to unsafe abortion, post-abortion care (PAC) was introduced for use in the management of incomplete abortion. This involves treating the affected women with empathy, uterine evacuation if incomplete, the use of antibiotics, and contraception among other components [4]. Originally, evacuation of the uterus in PAC focused on suction evacuation with high patronage of manual vacuum aspiration (MVA) in resource-poor settings. However, though MVA is a safe and effective treatment option for early pregnancy loss, [5] its accessibility in low resource countries is constrained by the unavailability of sterile equipment and skilled provider generally, [6] as well as other challenges such as the need for theatre space, cost of the procedure, and peculiar complications including cervical trauma and uterine perforation. Because of these constraints, there was a need to explore effective, accessible, and acceptable non-surgical options.

There is increasing evidence that misoprostol, a prostaglandin E2 analogue, is a safe, effective, and acceptable method to achieve uterine evacuation for women in need of PAC services because it does not require the immediate availability of sterilized equipment, operating theatres or skilled personnel [7]. These features among others render misoprostol an important alternative to PAC with MVA particularly in low resource and remote areas [8]. Misoprostol can be administered through several routes in obstetrics and gynecological practice, however, for medical management of incomplete abortion, a single oral dose 600mcg is recommended [9]. Very important to our resource-poor setting with the attendant under-developed health insurance system is the fact that misoprostol reduces the cost of post-abortion care services substantially by removing the need for theater space, sterile instruments, skilled personnel, and their associated financial implications. These advantages will be further enhanced by the identification of a more convenient route(s) of administration that allows a lower dose of misoprostol. Single-dose misoprostol 400mcg administered through the sublingual route appears to offer these enhanced advantages [10]. This route of administration and dosage are also associated with high levels of satisfaction and acceptability to patients involved [11]. In our setting, services and training on post-abortion care often focus on the use of MVA for uterine evacuation, [12] which calls for the scale-up of PAC services to include the use of misoprostol by the most convenient route and most effective small dose. This study, therefore, compared the efficacy of single-dose sublingual misoprostol 400mcg to manual vacuum aspiration for the complete evacuation of the uterus in cases of incomplete abortion in Enugu, South-east Nigeria. This study adhered to CONSORT guidelines in the reporting of the research findings.

## Methods

Study design: this study was an open-labeled randomized controlled study of 203 consenting women with incomplete abortion who presented at the gynecological emergency unit of the University of Nigeria Teaching Hospital (UNTH) Ituku-Ozalla, Enugu and Julius Memorial specialist Hospital Enugu, Nigeria. The UNTH is a teaching hospital that offers 24-hour emergency obstetrics care as part of its comprehensive multi-specialist emergency services. Julius Memorial Specialist Hospital is a private specialist hospital situated in Abakpa Nike- a suburb and the most densely inhabited part of Enugu metropolis. It runs a 24-hour comprehensive emergency obstetric care and serves as a referral center for maternity homes and health centers in Enugu East local government area of Enugu State, Nigeria. Enugu is the capital city of Enugu State which is in the South-eastern geopolitical zone of Nigeria.

Study setting: a teaching hospital, the University of Nigeria Teaching Hospital (UNTH) Ituku-Ozalla, Enugu and a private specialist hospital, Julius Memorial Specialist Hospital Enugu, Nigeria were used for the study.

Participants: two hundred and twelve (212) consecutive consenting women with sonologically confirmed, first-trimester incomplete spontaneous abortion in the gynaecological emergency departments of the study centers. Two hundred and three (203) study participants fulfilled the study requirements.

**Figure 1:**
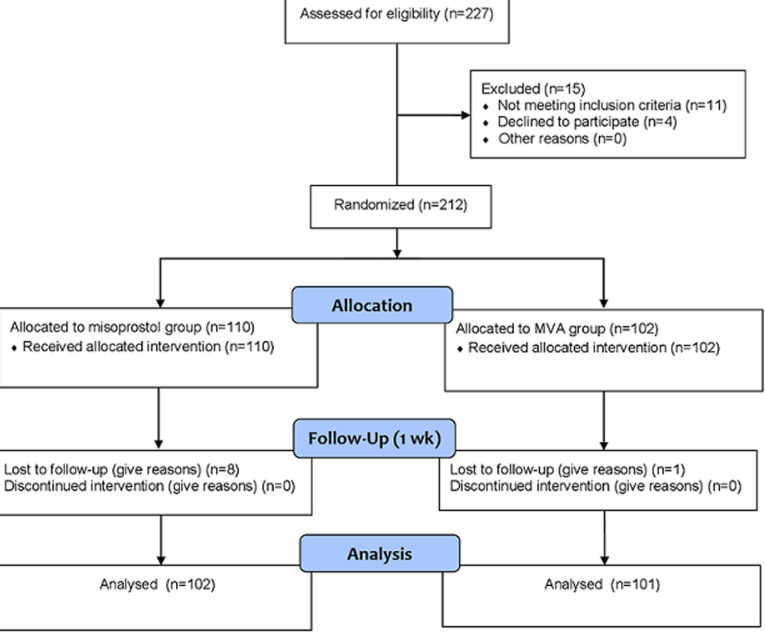
study flow chart

Sample recruitment: all consenting women at the study centers with first-trimester incomplete abortion, confirmed by a trans-abdominal ultrasound scan of the uterus, were eligible for the study. Women with excessive vaginal bleeding, severe anemia (Hb < 7.0g%), or suspected ectopic pregnancy were excluded from the study. Other exclusion criteria were a history of allergy to prostaglandins, evidence of genital infection such as offensive vaginal discharge, uterine tenderness, and pyrexia. As illustrated in Figure 1, 212 consecutive consenting eligible women were randomized into two treatment groups; intervention group A (n=110) received single-dose sublingual misoprostol 400mcg while the control group B (n=102) had immediate manual vacuum aspiration (MVA) of the uterus. Participants´ randomization was by simple random allocation - after obtaining informed consent, each participant was assigned a sequential number (1 to 212) by the principal researcher then, she picked one of the two balls (same size and texture) from a black bag kept with the research assistants. Each ball was marked A or B representing the two treatment groups. The participant was allocated to the treatment group written on the ball she picked. The sample size on each arm was adequate to identify a 95.5% difference in the incidence of complete uterine evacuation between the two treatment groups, based on the assumed incidence of complete abortion of 90% for the MVA (p_0_), power of 80%, alpha of 0.05, and attrition rate of 10%.

Data collection method: each participant in the misoprostol group was given 400mcg of misoprostol (Cytotec®) placed under their tongues for 30 minutes to enable the tablets to dissolve. They were observed for about 8 hours after the drug administration and allowed home. Each participant was given the mobile phone number of the principal investigator for the reporting of any side effects or complications. They were discharged on analgesics - oral ibuprofen 400mg twelve hourly for 5 days, and prophylactic antibiotics - oral amoxicillin/clavulanic acid 625mg 8 hourly, and metronidazole 400mg 8 hourly for 1 week. Follow-up at the gynecology clinic visit was scheduled for each participant after one week. During the follow-up visit, they were clinically and sonologically evaluated for complete abortion; according to the study protocol, those with incomplete uterine evacuation were offered immediate manual vacuum aspiration or further observation for an extra week after which surgical evacuation would be carried out if the uterine cavity was not empty. Participants in the MVA group (B) were offered immediate manual vacuum aspiration in the theater by trained medical practitioners, using standard procedure [13]. After the procedure, each was observed for 2 hours then, discharged home on the analgesics and prophylactic antibiotics described above. Each was scheduled for a follow-up clinic visit after 1 week of treatment, during which they were evaluated by ultrasound scan for complete uterine evacuation. In the case of incomplete uterine evacuation, the protocol was a repeat uterine evacuation by MVA.

At the initial treatment visit, participants´ baseline data on socio-demographics, gestational age, contraceptive history, and assigned treatment groups were documented. Their mobile phone numbers or that of a close relative was obtained. During the follow-up visit, information on the completeness of the uterine evacuation, treatment side effects and their severity, and satisfaction with treatment were obtained and recorded on the study´s datasheet for all participants. All documentations were made by the principal investigator and his trained assistants. Participant´s perceived severity of side-effect was assessed with a three-point Likert scale (i.e. mild/tolerable, moderate/less tolerable, and severe/intolerable). Also, the participants´ satisfaction with the treatment received was assessed using the visual analogue scale (VAS) (0 - 100), as described by Mba et al. [14]. All participants were also offered post-abortion contraceptive counseling and service and linked to other reproductive health services as appropriate. For the study, incomplete abortion was diagnosed when a pregnant woman presented with a history of vaginal bleeding with dilated cervix on physical examination as well as abdominopelvic ultrasound scan features of a retained product of conception.

Variables: the primary outcome measure was the incidence of complete uterine evacuation (complete abortion) after one week of treatment with single-dose sublingual misoprostol 400mcg or MVA. The secondary outcome measures included incidence, types, and tolerability of treatment side effects as well as participants´ satisfaction with the treatment received.

Ethical approval and consent to participate: the ethical clearance certificate number NHREC/05/01/2008B - FWA00002458 - IRB 00002323 for this research was obtained from Research and Ethics Committee of University of Nigeria Teaching Hospital Ituku/ Ozalla, Enugu. A written informed consent was obtained from each participant before they were recruited into the study.

Data analysis: data for 102 and 101 women were analyzed in the intervention (misoprostol) and control (MVA) groups respectively (Figure 1) using the statistical package for social sciences (SPSS) computer software, version 20.0 for Windows (IBM Corporation). Data analysis was per protocol. Continuous variables were compared using the student´s t-test while the discrete variables were analyzed using proportions, and their associations compared with Pearson´s chi-square or Fisher´s exact where applicable. A probability value of less than 0.05 was considered statistically significant.

## Results

Out of a total of 227 participants assessed for eligibility, 212 participants met the study criteria. At randomization 110 received the intervention while 102 were in the control group. A total of eight and one participants in the intervention and control groups respectively were lost to follow up. Therefore, a total of 203 participants who met the study criteria and completed the study from August 2014 to February 2015 were randomised into the intervention group (n = 102) that received single-dose sublingual misoprostol 400mcg and the control group (n = 101) that received manual vacuum aspiration. Details were shown in Figure 1. The study ended when the required sample size was completely recruited and followed-up. Participants' basic characteristics (Table 1) were similar between the two groups. The mean ages of the participants were 28.7 ± 5.83 versus 29.0 ± 6.49 years for the misoprostol and MVA groups respectively (p = 0.795). Likewise, the mean gestational age at presentation was 9.1 ± 2.0 weeks for the misoprostol group and 9.1 ± 2.1 weeks for the MVA group (p = 0.975). The incidence of complete uterine evacuation after 7 days of follow-up was 86.3% (88/102) for the misoprostol group and 100.0% (101/101) for the MVA group.

**Table 1:** basic characteristics of the study participants

Characteristics	Sub-groups	Misoprostol group (n = 102)	MVA group (n= 101)	P- value
		Frequency (%)	Frequency (%)	
Age (years)	20	1 (1.0%)	4 (4.0%)	0.219
	20-24	29 (28.4%)	28 (27.7%)	
	25-29	25 (24.5%)	19 (18.8%)	
	30-34	29 (28.4%)	32 (31.7%)	
	35-39	14 (13.7%)	23 (11.3%)	
	40-44	4 (3.9%)	9 (8.9%)	
	Mean ±SD	28.7± 5.83	29.0 ± 6.49	0.795
Marital status	Married	75 (48.7%)	79(51.3%)	0.435
	Single	27(55.1%)	22(44.9%)	
Educational status	Primary	1 (20.0%)	4 (80.0%)	0.260
	Secondary	43 (47.8%)	47 (52.2%)	
	Tertiary	58 (53.7%)	50 (46.3%)	
Parity groups	0	31 (54.4%)	26 (45.6%)	0.634
	1	14 (43.8%)	18 (56.2%)	
	2- 4	52 (51.5%)	49 (48.5%)	
	≥5	5 (38.5%)	8 (61.5%)	
	Mean ±SD	1.8 ± 1.53	2.0 ±1.78	0.378

The observed difference was statistically significant RR = 0.86, (CI 95%: 0.80 - 0.93), p <0.001. All women with incomplete uterine evacuation after 7 days of treatment in the misoprostol group (14/102, 13.7%), opted for MVA of the uterus rather than an extra week follow-up for the misoprostol effect. Details of the types and distribution of side effects among study participants are shown in Table 2. Treatment side effects were commoner in the misoprostol group (88.2% (90/102) compared to the MVA group (57.4%, 58/101), RR = 1.5, (CI 95%: 1.28, 1.84), p < 0.001. The most common side effect was abdominal pain which had an incidence of 27.5% (28/102) in the misoprostol group versus 48.5% (49/101) in the MVA group, RR = 0.6, (CI95%: 0.39 - 0.82), p = 0.002. As shown in Table 3, most participants with side effects in both groups considered them as mild and tolerable - misoprostol group (81.1%, 73/90) versus (77.6% 45/58), RR = 1.1 (CI95%: 0.88 - 1.24), p= 0.677. As regards to maternal satisfaction for the treatment received, the mean VAS scores for the misoprostol group (86.7 ± 14.11) were significantly higher than that of the MVA group (81.36 ± 11.10), p < 0.001. Eighty (88.9%) participants in the misoprostol group would recommend the treatment to other women with incomplete abortion in the first trimester while 63 (62.4%) women in the control group would recommend MVA to women in a similar situation, RR = 1.3, (CI 95%: 1.05 - 1.51), p = 0.014.

**Table 2:** side effects distribution by treatment groups

Side effect	Misoprostol (n= 102)	MVA (n = 101)	P value	RR (CI 95%)
	Freq (%)	Freq (%)		
Abdominal pain	28 (27.5)	49 (48.5)	0.002	0.6 (0.39, 0.82)
Nausea	16 (15.7)	1 (1.0)	< 0.001	15.8 (2.14, 117.24)
Vomiting	18 (17.7)	3 (3.0)	< 0.001	5.9 (1.81, 19.55)
Diarrhoea	10 (9.8)	0 (0.0)	< 0.001	-
Bleeding	6 (5.9)	1 (1.0)	0.119	6.0 (0.73, 48.47)
Chills	12 (11.8)	4 (4.0)	0.654	3.0 (0.99, 8.90)
All side effects	<="" td="" style="padding: 7px; border: 1px solid rgb(221, 221, 221); border-collapse: collapse;">	58 (57.4)	< 0.001	1.5 (1.28, 1.84)

**Table 3:** tolerability of treatment’ side effects

Side effects	Severity	Misoprostol (n = 90)	MVA (n= 58)	P - value	RR (CI 95%)
		Freq (%)	Freq (%)		
Tolerable	Mild	73 (81.1)	45 (78.6)		
Yes	Moderate	15 (16.9)	12 (20.7)	0.677	1.1 (0.88 - 1.24)
No	Severe	2 (2.2)	1 (1.7)	-	-

## Discussion

It is recognized that about 10 - 15% of all pregnancies end in spontaneous abortion, [15] and another proportion is willfully terminated due to unintended pregnancy; for instance, rates of 33 to 46 induced abortions per 1,000 women of reproductive age group were reported in Nigeria [16,17]. It is noteworthy that either of these categories can end in incomplete abortion with the attendant morbidities and mortality when not managed appropriately. Thus, the need for a more convenient, affordable, and non-surgical treatment method in our environment cannot be over-emphasized. This study has shown that treatment of incomplete abortion with a single dose sublingual misoprostol 400mcg followed up for 1 week was effective for the complete abortion though, it was about 14% less effective than immediate uterine evacuation with MVA. The finding in the misoprostol group of this study is similar to 88.4% reported in Ibadan, Nigeria, [6] and 86.9% in Burkina Faso [18]. However, it is lower than 91.8% reported in a multi-country study from sub-Saharan Africa, [10] 98.3% in Egypt, [11], and 99.4% in Senegal [19]. All these related studies reviewed the misoprostol treatment success rate after 7 days´ follow-up of participants. As demonstrated in earlier studies, [6, 18] a follow-up of participants for two weeks after misoprostol administration yields higher uterine complete evacuation rate of up to 98.0%, [18] which is comparable to the MVA evacuation rate in this study and other studies [10,11,20]. This might explain the recommendation that women being treated with misoprostol as part of post-abortion care should be followed-up for 1 to 2> weeks to ensure complete abortion [2]. Unfortunately, this study has no data for 2 weeks follow-up because participants in the misoprostol group with incomplete evacuation after 1 week of treatment, did not consent to an extra week of follow-up.

The study found that the treatment side effects were commoner in the misoprostol group but, fortunately, over 80% of the complaints were mild in severity, which might explain the lack of difference in the side effect tolerability by participants in the two treatment groups. Previous studies had also found no difference in the tolerability of side effects between the two treatment groups [10,11]. Misoprostol for uterine evacuation works through its uterotonic property so, a higher incidence of abdominal pain, as a side effect, is expected among participants in that group, [10,11] but that was not the case in this study for reasons that are not clear. A related study of a similar population in Nigeria observed more abdominal pain in the misoprostol group (single-dose oral misoprostol 600mcg) when compared to the MVA group, [21] which might rule-out population peculiarities as a possible reason for this unusual finding. Though a report from Madagascar found no difference in the mean abdominal pain score between cohorts of women that used oral (600mcg) versus sublingual (400mcg) misoprostol for incomplete miscarriage treatment, [22] it is important to explore in subsequent studies whether the sublingual route of misoprostol is associated with less abdominal pain in our environment.

Misoprostol treatment was highly acceptable to participants in this study when compared to the MVA considering that they were more likely to recommend the treatment to other women with incomplete miscarriage when compared to women that had MVA. This high acceptance was also expressed in related studies [6,10,18]. This study did not seek reasons for this high acceptability but, it was likely to border on the fact that the treatment did not involve the use of surgery so, it was viewed as more convenient and cheaper. It has been established that women from the study environment have a strong aversion for cesarean sections, [23,24] and it may not be out of place to believe that this aversion extends to other pregnancy-related surgeries including MVA. Future studies in this subject should specifically seek for reason for the preference/non-preference of each treatment method. The nature of the study made blinding of both investigators and participants difficult but, the lack of blinding could not have impacted the study´s estimates. Discontinuation rate when assessed as an outcome measure might reflect adverse events; [25] it is, therefore, likely that the larger loss to follow-up in the misoprostol group might be related to severe treatment side effect; however, further data analysis showed that this scenario did not affect the treatment tolerability in both groups. The study remains relevant in Nigeria and similar under-resourced settings because it has added strong evidence for the use of a lower and cheaper dose of misoprostol through a convenient route, for the treatment of incomplete abortion.

Trial registration: the study trial registration number, PACTR202009857889210 was registered retrospectively with the Pan African Clinical Trial Registry database. Date of registration - 23 September 2020.

## Conclusion

This study has shown that the use of single-dose sublingual misoprostol 400mcg followed up for 1 week, for the treatment of incomplete abortion produced a high rate of complete abortion though, it was less effective than surgical evacuation by manual vacuum aspiration. Also, women that received single-dose sublingual misoprostol 400mcg had a higher incidence of side effects when compared to the MVA group but, the majority of the side effects were mild and tolerable. Finally, when compared to women that received MVA, women treated with misoprostol were more satisfied with their treatment and were more likely to recommend the treatment to others with incomplete first-trimester abortion. It is therefore recommended that the single-dose misoprostol 400mcg should be included as a treatment option for the post-abortion care of eligible women with first-trimester incomplete abortion in the study environment.

### What is known about this topic

- Evacuation of the uterus in post abortion care focused on suction evacuation with high patronage of manual vacuum aspiration (MVA) in resource-poor settings;
- MVA is a safe and effective treatment option for early pregnancy loss and its accessibility in low resource countries is constrained by the unavailability of sterile equipment and skilled providers generally as well as other challenges such as the need for theatre space, cost of the procedure, and peculiar complications including cervical trauma and uterine perforation;
- In low resource setting, services and training on post-abortion care often focused on the use of MVA for uterine evacuation, which calls for the scale-up of PAC services to include the use of misoprostol by the most convenient route and most effective small dose.

### What this study adds

- Single-dose sublingual misoprostol 400mcg followed up for 1 week, for the treatment of incomplete abortion produced a high rate of complete abortion that was not statistically different from that of surgical evacuation by manual vacuum aspiration;
- Women treated with misoprostol were more satisfied than women that received manual vacuum aspiration as their treatment and were more likely to recommend the treatment to others with incomplete first-trimester abortion.
